# Enhancing inherent soil productivity increases maize yield and nitrogen use efficiency by improving soil water and nutrient status

**DOI:** 10.3389/fpls.2026.1816672

**Published:** 2026-04-14

**Authors:** Zhipeng Cheng, Meiren Rong, Yajian Li, Fugui Wang, Zhen Wang, Yongqiang Wang, Ranran Guo, Lanfang Bai, Zhigang Wang

**Affiliations:** 1 College of Agronomy, Inner Mongolia Agricultural University, Hohhot, China; 2 Inner Mongolia Autonomous Region Industrial Technology Engineering Center for Intelligent Water and Fertilizer Management Technology and Equipment for Crops, Hohhot, China; 3 Institute of Biotechnology, Tongliao Academy of Agricultural and Animal Husbandry Sciences, Tongliao, China

**Keywords:** inherent soil productivity, maize, nitrogen use efficiency, soil nutrients, yield

## Abstract

**Introduction:**

Soil management technologies centered on fertility enhancement are essential for achieving the synergistic improvement of crop yield and resource use efficiency. This study aims to identify key soil factors driving the coordinated increase in yield and nitrogen use efficiency of spring maize, thereby providing a scientific basis for promoting high yield and efficiency through improved soil quality.

**Methods:**

Based on variations in inherent soil productivity (ISP) across maize ecological regions in Inner Mongolia, China, this study established two soil management modes—Conventional Practices (CP) and Improved Soil Management Practices (IMSP)—to quantitatively identify key factors influencing ISP, yield and nitrogen use efficiency (NUE) through the random forest algorithm. The effects of soil fertility improvement on maize yield and nitrogen use efficiency were also examined.

**Results:**

Our results showed that soil organic matter and alkali-hydrolysable nitrogen were the major nutrient related drivers of ISP, maize yield, and NUE, whereas N mineralization, N losses, soil water content, and WUE dominated process-related regulation. Among these, soil organic matter was the core factor governing ISP. Soil organic matter content indirectly affects soil nitrogen mineralization and water conservation capacity, which is the core of regulating soil productivity. The core process of synergistic regulation of maize yield and NUE by fertility improvement mainly lies in two aspects: on the one hand, by expanding the storage capacity of soil water to meet the water demand of the maize population; on the other hand, by increasing the net mineralization of soil nitrogen (16.0%), reduce the apparent loss of nitrogen (11.4%), reduce the risk of nitrogen leaching, and make it have the best soil water and nitrogen environment, so as to achieve synergy between yield and NUE. The regulation effect of IMSP model and nitrogen-dense interaction on yield increase and efficiency is more significant.

**Discussion:**

Enhancing soil fertility through increased organic matter improves water storage and net nitrogen mineralization while reducing nitrogen loss. The IMSP model and nitrogen-dense interaction effectively regulate this process, creating an optimal soil environment to synergistically achieve high maize yield and improved nitrogen use efficiency.

## 1 Introduction

The mismatch between the supply capacity of the tillage layer and crop demand is a major constraint to achieving both high yield and high efficiency in maize production. In the face of the country’s demand for grain increase, how to overcome the problem of “increasing production can only rely on water and fertilizer input” has become an important research task at present (Maitra et al., 2021). Some scholars have tried to solve this problem from the perspective of soil productivity. The study believes that the contribution rate of inherent soil productivity to the increase in maize yield since the 1980s is 22%, and under the same cultivation measures, with the improvement of the level of soil, maize yield has also risen (Fan et al., 2013). Through the optimization of cultivation measures to create different soil base productivity, the results show that when the inherent soil productivity is increased to 8.0 t ha^-1^ or more, the maize yield and NUE can be improved in synergy, and the contribution rate of soil to yield can be stabilized at 80% (Wang et al., 2022). It can be seen that, given the current limited arable land resources and limited N fertilizer inputs, improving the basic productivity of the soil can lead to higher yields with less fertilizer input. However, inherent soil productivity (ISP) is a comprehensive indicator, often affected by the combination of soil physical, chemical, biological, water and heat characteristics (Zhang, 2023). Therefore, it is necessary to quantitatively analyze the core limiting factors of inherent soil productivity and their interaction mechanism with maize yield and NUE, which is of great significance for relying on soil power improvement to achieve high yield, high efficiency and environmentally friendly synergy of maize.

From an ecological point of view, the fundamental reason why inherent soil productivity affects maize yield and NUE lies in the difference in soil characteristics (Xin et al., 2016). Some scholars have studied the effect of soil chemistry on yield. The main factors limiting high yielding fields as soil organic matter, effective phosphorus and fast-acting potassium, and believed that high-yield fields can better utilize and transform organic nutrients such as soil carbon, nitrogen and phosphorus, thereby promoting soil carbon sequestration to achieve high yields (Zhang et al., 2009). Soil organic matter, available nitrogen, and available potassium have been identified as key nutrient factors affecting inherent soil productivity (Zhao et al., 2016). In addition, soil structural constraints are major factors limiting water and fertilizer use efficiency (Lipiec et al., 2012). Existing studies on soil water conservation and moisture enhancement have mainly focused on optimizing tillage practices in combination with exogenous organic matter inputs to improve soil structure and water-holding capacity, thereby enhancing the coordination between soil moisture and nutrient availability and ultimately improving soil water and fertilizer supply capacity (Demir and Demir, 2019; Soinne et al., 2023; Liu et al., 2024). From the perspective of soil nitrogen dynamics, total nitrogen, inorganic nitrogen, and mineralized nitrogen are considered decisive factors in maize yield formation and nitrogen uptake (Shi et al., 2022). Moreover, optimized water and nitrogen management can not only improve water and nitrogen use efficiency, but also reduce nitrogen losses significantly (Iglesias et al., 2025; Shi et al., 2023; Menegaz et al., 2022; Yang et al., 2024). High-fertility farmland generally provides a more favorable soil water and nitrogen environment, which helps reduce deep percolation losses of soil water, enhance soil nitrogen mineralization capacity, and consequently decrease nitrogen loss (Zhou, 2023).

The above-mentioned studies have well answered the characteristics of soil water and nitrogen under high fertility farmland, but there is no clear conclusion on the underlying causes of soil water and nitrogen environmental improvement affected by the improvement of inherent soil productivity. To answer this question, in-depth research is needed from the characteristics of soil structure, nutrient status and water use. In this study, we selected maize planting ecological areas with great fertility differences in Inner Mongolia, created different inherent soil productivity through different soil management modes combined with nitrogen dense interaction, and compared and analyzed soil nutrient status, water use and nitrogen mineralization characteristics.

Therefore, two key scientific questions were proposed: (1) What are the quantitative contributions of different soil factors to inherent soil productivity? and (2) What are the soil regulatory pathways through which enhanced inherent soil productivity promotes the synergistic improvement of maize yield and nitrogen use efficiency? Through random forest analysis, the core factors limiting inherent soil productivity and their synergistic regulation mechanism on yield and NUE were quantified and analyzed, so as to provide a theoretical basis for exploiting the potential of soil production to achieve maize yield and efficiency.

## 2 Materials and methods

### 2.1 Experimental site and experimental materials

Field experiments were conducted from 2015 to 2016 across five representative ecological zones of Inner Mongolia: The Hilly Region of Southern Greater Khingan, the West Liao River Plain, the Hilly Region of Northern Yan Mountains, the Tumed Plain, and the Hetao Plain. At all sites, maize was the preceding crop, and conventional management practices were adopted, including shallow rotary tillage without crop residue incorporation or manure application.

Detailed information on the climatic conditions, farmland soil types, and soil fertility status of each experimental site is presented in the **Supplementary Materials** (**Supplementary Table S1**). Considering the variability in thermal conditions that could influence maize growth, a uniform hybrid, Pioneer 335, was planted at each location. To minimize the confounding effect of water stress caused by uneven precipitation, micro-sprinkler irrigation was applied only when transient leaf wilting occurred.

### 2.2 Experimental design

At each site, a three-factor factorial experiment (2 × 3 × 2) was established, comprising two soil management strategies [conventional practices (CP) and improved soil productivity (IMSP)], three plant densities (60,000, 82,500, and 105,000 plants ha⁻¹), and two nitrogen rates (0 and 220 kg ha⁻¹). Adjacent to the CP plots, additional unfertilized controls were included to estimate the inherent soil productivity (ISP) at each plant density.

The CP strategy represented local smallholder practices, involving shallow rotary tillage (15 cm depth) without residue incorporation or manure application. The IMSP treatment aimed to enhance ISP by increasing soil organic matter (SOM) by approximately 10 g kg⁻¹ through the application of 79.5 t ha⁻¹ of composted sheep manure (30% organic matter), combined with maize residue return and deep tillage to 35 cm. In the following spring, all plots were prepared using shallow rotary tillage (10 cm) to consolidate the seedbed. Inherent soil productivity (ISP) for each ecological region was estimated from the mean maize yield across different planting densities in unfertilized control plots under the CP management mode. The average yield and corresponding ISP values for each ecological region during 2015–2016 are provided in the **Supplementary Materials** (**Supplementary Table S2**).

At each location, the factorial experiment was arranged in a split–split plot design with three replicates. Soil management strategy was assigned to the main plots, plant density to the subplots, and fertilizer rate to the sub–subplots. Each sub–subplot had a planting area of 86.4 m². At sowing, all treatments except the unfertilized control received 46 kg P ha⁻¹ (as triple superphosphate) and 37 kg K ha⁻¹ (as potassium sulfate), band-applied 5 cm below and 5 cm beside the seed row. For the 220 kg N ha⁻¹ treatment, nitrogen (as urea) was side-dressed at the six-leaf stage, placed 10 cm below and 10 cm from the plant row to minimize seedling injury and reactive N losses (Zhang et al., 2009). Effective weed control was achieved through pre- and post-emergence herbicide applications, while pesticides and fungicides were applied by unmanned aerial vehicles when necessary. Maize yields for all treatments at each site are presented in **Supplementary Table S2**.

### 2.3 Sampling and measurement

#### 2.3.1 Soil nutrient

Before land preparation, composite soil samples (0–30 cm depth) were collected from three points in each plot using a soil auger. The samples were analyzed for soil organic matter (SOM; organic C determined by wet digestion with H_2_SO_4_–K_2_Cr_2_O_7_ and converted to SOM by multiplying by 1.724), alkali-hydrolyzable nitrogen, Olsen phosphorus (Olsen-P), and available potassium (Available K) (Bao, 2000).

#### 2.3.2 Apparent N loss from soil

Soil sampling was conducted before sowing and after harvest using a five–point composite method at 0–100 cm depth. The soil profile was divided into four layers: 0–20, 20–40, 40–70, and 70–100 cm. Samples from each depth were homogenized and sealed in Ziplock bags. Inorganic nitrogen concentrations were analyzed using a continuous flow injection analyzer (AA3HR, Germany) to estimate soil residual nitrogen and apparent nitrogen loss (Hartmann et al., 2014). The specific calculation formulas are shown in Equations 1–3:

(1) $$N\;min=d\times P_{b}\times C\times 0.1$$

(2) $$N_{loss}=N_{rate}+Pre\;N_{min}+Min\;N–I_{t}–Harvest\;N_{min}$$

(3) $$Min\;N=(I_{0}+Harvest\;N_{0}min)–Pre\;N_{0}min$$

where *N_min_* is the cumulative amount of soil nitrate–N and ammonium–N (kg ha⁻¹); *d* denotes soil layer thickness (cm); *P_b_* is bulk density (g cm⁻³); *C* represents the concentration of nitrate–N or ammonium-N (mg kg⁻¹); and 0.1 is the unit conversion factor. *N_loss_* indicates the apparent nitrogen loss (kg ha⁻¹); *N_rate_* is the nitrogen fertilizer application rate (kg ha⁻¹); *Pre N _min_*, *Min N*, and *Harvest N _min_* correspond to pre–sowing soil mineral N, soil mineralized N, and post–harvest soil mineral N (kg ha⁻¹), respectively. *I_t_* refers to crop N uptake (kg ha⁻¹). *I_0_*, *Pre N_0 min_*, and *Harvest N_0 min_* represent maize nitrogen uptake, pre–sowing, and post-harvest soil mineral N under the unfertilized control (kg ha⁻¹), respectively. The cumulative soil mineral N represents the total amount of nitrate–N and ammonium–N throughout the soil profile.

#### 2.3.3 Maize nitrogen concentration

At the physiological maturity stage (R6), three uniformly grown maize plants were collected consecutively from each treatment plot. The plants were divided into four components: (1) leaves, (2) stems (including stalks, leaf sheaths, and tassels), (3) ears (including cobs, husks, and ear shanks), and (4) kernels. All samples were oven–dried following a two-step protocol: first at 105 °C for 30 min to deactivate enzymes, and then at 80 °C until constant weight. The dry weights were recorded for biomass determination, after which samples were ground to pass through a 1 mm sieve. Total nitrogen concentration in each component was analyzed using the Kjeldahl method. Calculations related to NUE are shown in Equation 4:

(4) $$NUE=(Y_{t}–Y_{0})/N_{rate}$$

Where NUE represent nitrogen use efficiency (kg kg⁻¹); *Y_t_* and *Y_0_* represent maize yields (kg ha⁻¹) under the treatment and the nitrogen–free control, respectively; N_rate_ is the nitrogen application rate (kg ha⁻¹).

#### 2.3.4 Water use efficiency

Soil water content in each 20 cm layer within the 0–100 cm soil profile was measured before sowing, at the silking stage, and at maturity using a time domain reflectometer (TRIME-IPH, Germany). Maize water use efficiency (WUE) was calculated as follows (Guo et al., 2015; Wang et al., 2019). The specific calculation formulas are shown in Equations 5–7:

(5) $$WUE=Y_{t}/ET$$

(6) ET=P+I+ΔSWS

(7) $$SWS=\sum_{i=1}^{5}(h_{i}\times a_{i}\times \theta i)\times 10$$

Where *WUE* denotes water use efficiency (kg ha^-1^ mm^-1^); *Y_t_* represent maize yields (kg ha⁻¹); *ET* represents the water consumption of maize during the growing season (mm); *P* represents the precipitation during the maize growing season(mm); *I* represent the irrigation amount(mm); *△SWS* represents the difference in soil water storage within the 0–90 cm layer between pre-sowing and post-harvest(mm); *SWS* represents soil water storage(mm), *h* is the soil layer thickness (cm), *α* is the soil bulk density (g cm⁻³), *θ* is the soil water content (%), *i* denotes the soil layer, and 10 is the unit conversion coefficient.

### 2.4 Statistical analysis

All statistical analyses were conducted using SPSS 22.0 (SPSS Institute Inc., Chicago, IL, United States). Differences among treatments were evaluated by one-way ANOVA, and mean comparisons were performed using the least significant difference (LSD) test at *P* < 0.05. Linear regression analysis was used to examine relationships between variables, with significance determined by the corresponding P-values. Figures were prepared using SigmaPlot 12.5 (Systat Software Inc., San Jose, CA, United States). A random forest model was employed to quantitatively assess the importance of factors influencing changes in ISP, yield, and NUE. (R version 4.3.1; R Foundation for Statistical Computing, Vienna, Austria).

## 3 Results

### 3.1 Key soil factors affecting ISP, maize yield and NUE

**Figure 1:**
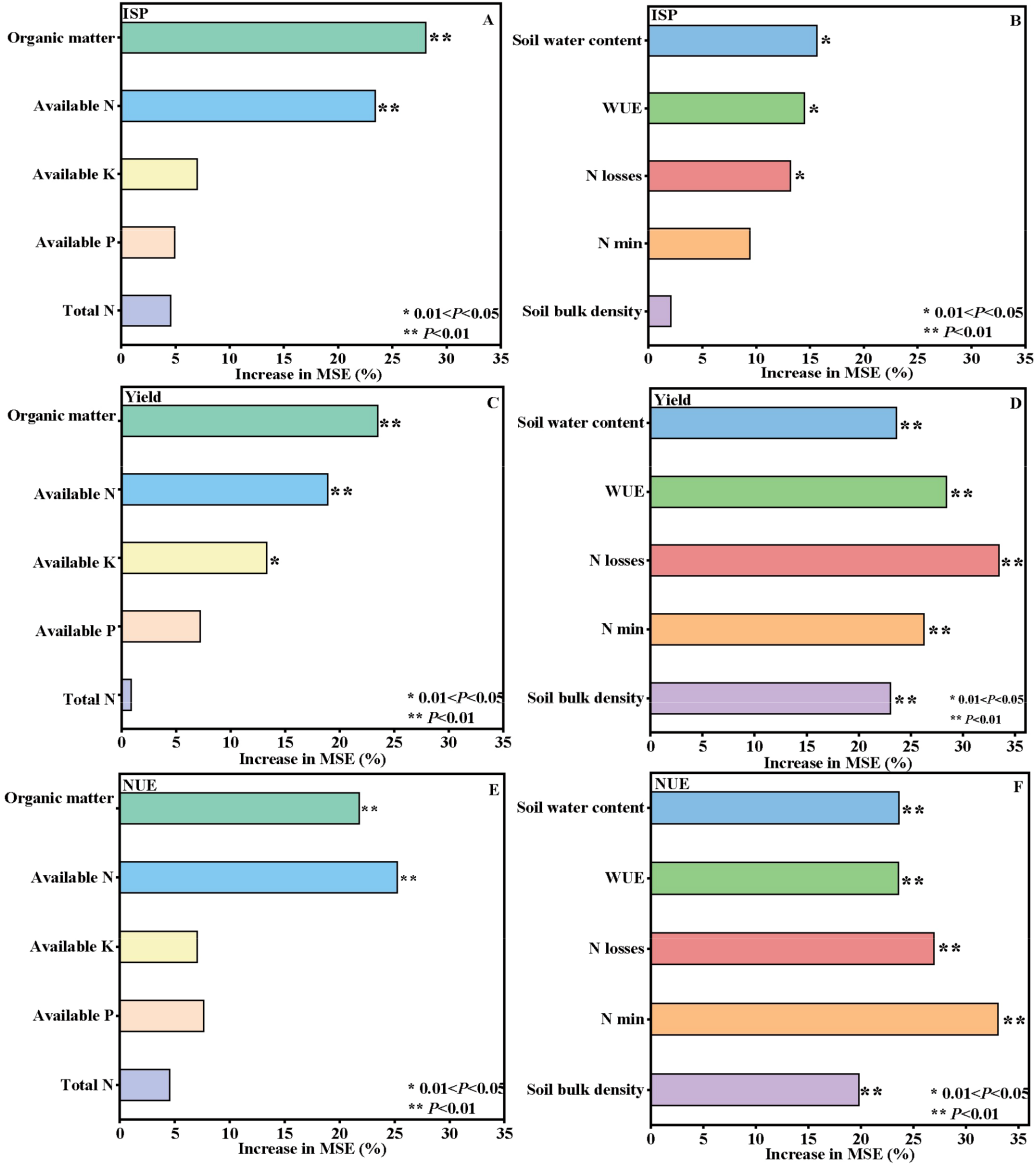
Contribution rate of soil factors to ISP, yield and NUE of maize. *0.01<*P*<0.05; **P<0.01.

Random forest analysis was performed to assess the relative importance of soil physical and chemical properties in regulating ISP, maize yield, and NUE (**Figure 1**). In terms of soil nutrient attributes, soil organic matter and alkali-hydrolyzable nitrogen were identified as the primary determinants of ISP, contributing 28.1% and 23.4%, respectively (**Figure 1A**). Regarding soil N mineralization, physical structure, and water-related processes, soil water content, WUE, and N losses were the dominant factors controlling ISP (**Figure 1B**). For maize yield, soil organic matter, alkali-hydrolyzable nitrogen, and available potassium were the main controlling variables (**Figure 1C**). In addition, soil water content, WUE, N losses, N min, and soil bulk density all exerted highly significant effects on maize yield, among which N losses, WUE, and N min showed the greatest contributions, accounting for 33.5%, 28.4%, and 26.3%, respectively (**Figure 1D**). For NUE, soil organic matter and alkali-hydrolyzable nitrogen were the most important nutrient-related indicators (**Figure 1E**). Meanwhile, soil water content, WUE, N losses, N min, and soil bulk density also had highly significant effects on NUE. Among these variables, N min, N losses, and soil water content contributed the most, with contribution rates of 33.0%, 29.9%, and 26.9%, respectively (**Figure 1F**).

### 3.2 The relationship between inherent soil productivity and soil nutrients

**Figure 2:**
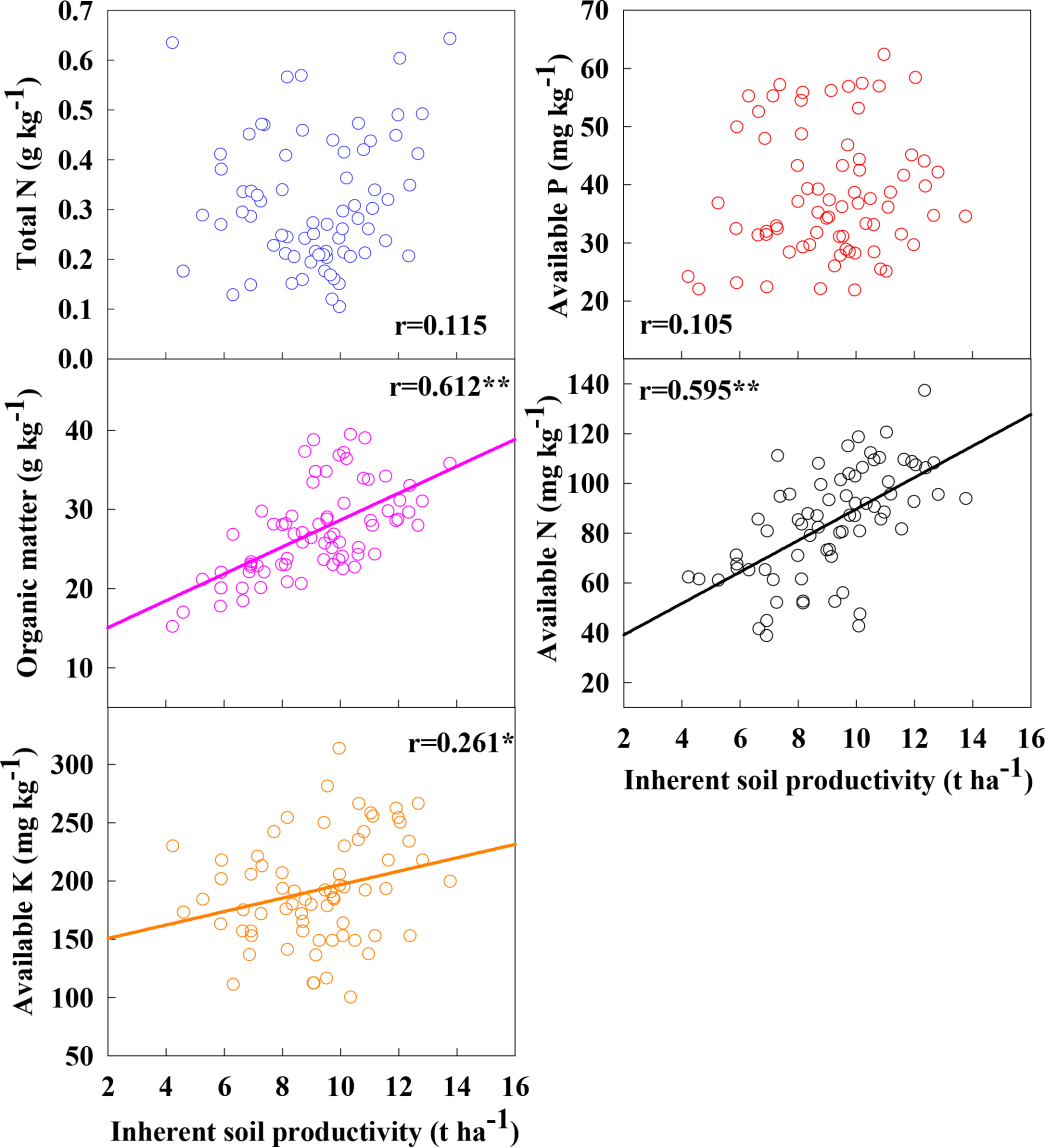
Correlations between inherent soil productivity and soil ingenious fertility. * and ** indicate significant differences at P < 0.05 and P < 0.01, respectively.

**Figure 3:**
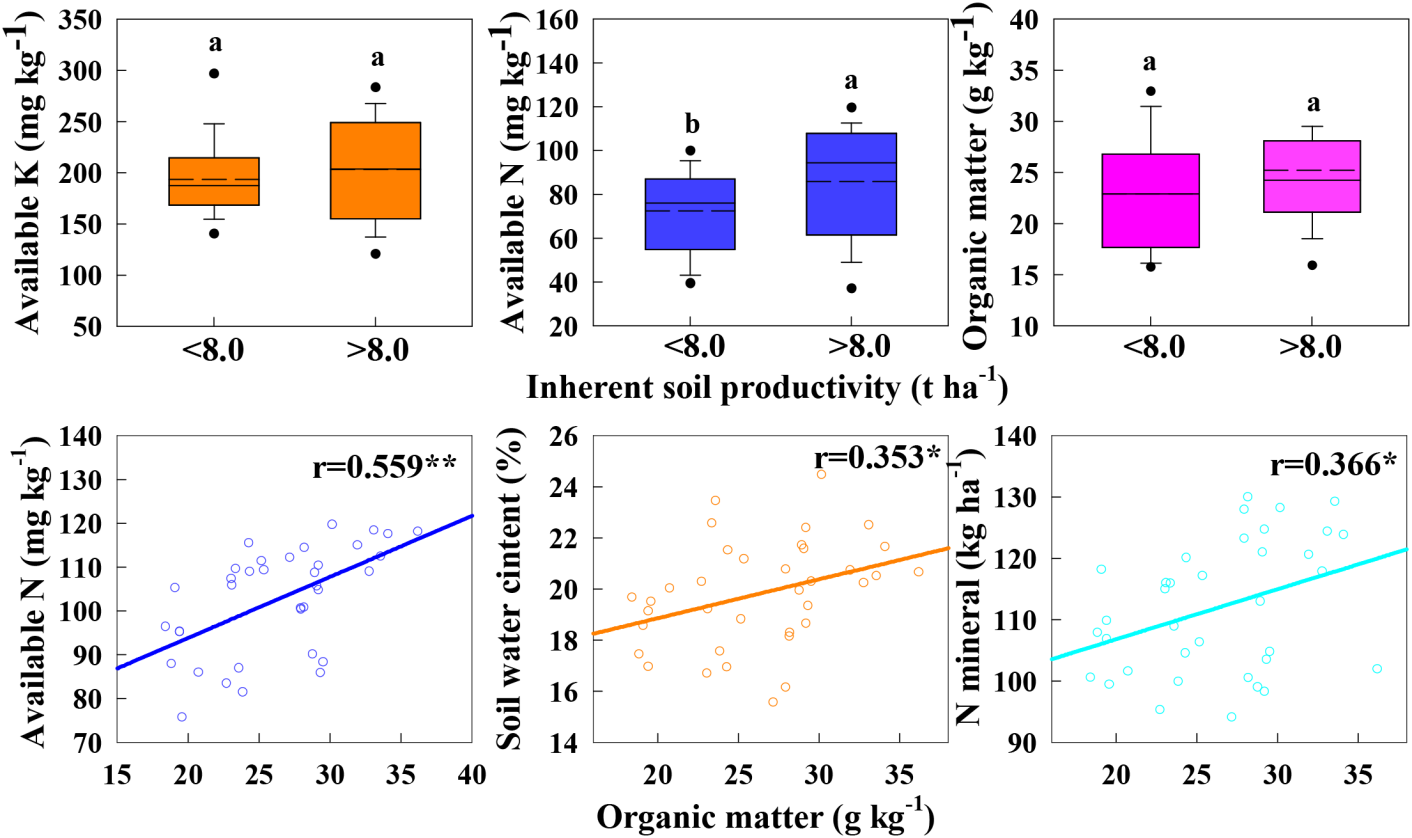
Correlations between organic matter and the other key factor. * and ** indicate significant differences at P < 0.05 and P < 0.01, respectively.

The correlation between soil nutrient factors and inherent soil productivity in different ecological areas was analyzed (**Figure 2**). The results showed that inherent soil productivity was significantly positively correlated with soil organic matter and alkaline hydrolysis nitrogen (r=0.595**~0.612**), and significantly positively correlated with available potassium (0.261*). Based on the results of Wang et al., the ISP of 8.0 t ha^-1^ was used as the boundary to compare the nutrients of soils with ISP lower than 8.0 t ha^-1^ and higher than 8.0 t ha^-1^ (**Figure 3**) (Wang et al., 2022). It was found that only alkaline hydrolysis nitrogen was significantly different at these two ISP levels, indicating that the supply capacity of soil available nitrogen was the key factor regulating ISP. From the box diagram in **Figure 3**, it is further found that the overall lower limit value of soil organic matter content with ISP higher than 8.0 t ha^-1^ is significantly higher than that of soil organic matter below 8.0 t ha^-1^, that is to say, soil organic matter content also has a decisive effect on ISP. The correlation analysis between soil organic matter and other soil main controlling factors also showed that the relationship between soil organic matter and alkaline hydrolysis nitrogen was significantly positive (r=0.559**), and the relationship between soil organic matter and soil water content and nitrogen mineralization was significantly positive (r=0.353*~r=0.366*). It shows that increasing soil organic matter content can indirectly affect soil available nitrogen, nitrogen mineralization capacity and water conservation capacity, thereby increasing ISP.

### 3.3 Effects of different soil management modes and densities on soil bulk density

**Figure 4:**
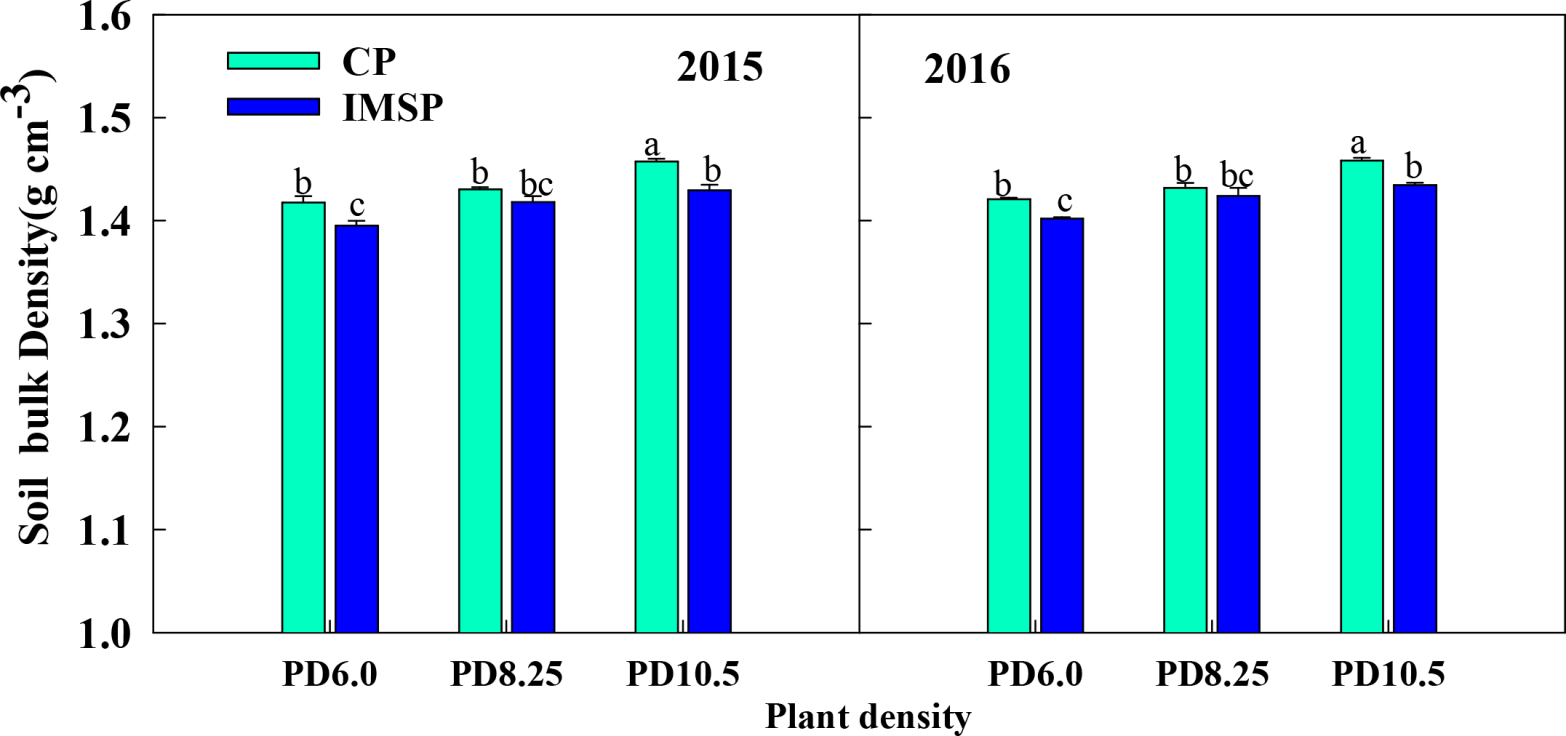
Comparison of soil bulk density under different soil management practices and plant densities at physiological maturity in 2015 and 2016 study year. Different lowercase letters indicate significant differences among treatments at P < 0.05.

Soil bulk density is an important index to characterize soil physical environment and resource status (Maitra et al., 2021). The test results in 2015 and 2016 showed that (**Figure 4**), the IMSP mode significantly reduced the soil bulk density, and the average difference was 1.4% compared with the CP mode. The soil bulk density increased slightly after increasing the density, but there was no significant difference in bulk density between different densities under IMSP mode, and the gain between high and low densities (0.7%-2.2%) was lower than that of CP mode (1.9%-2.7%). It shows that the interaction between IMSP mode and density can loosen soil structure and enhance soil permeability and water retention capacity.

### 3.4 Effects of different soil management modes and densities on soil water content

**Figure 5:**
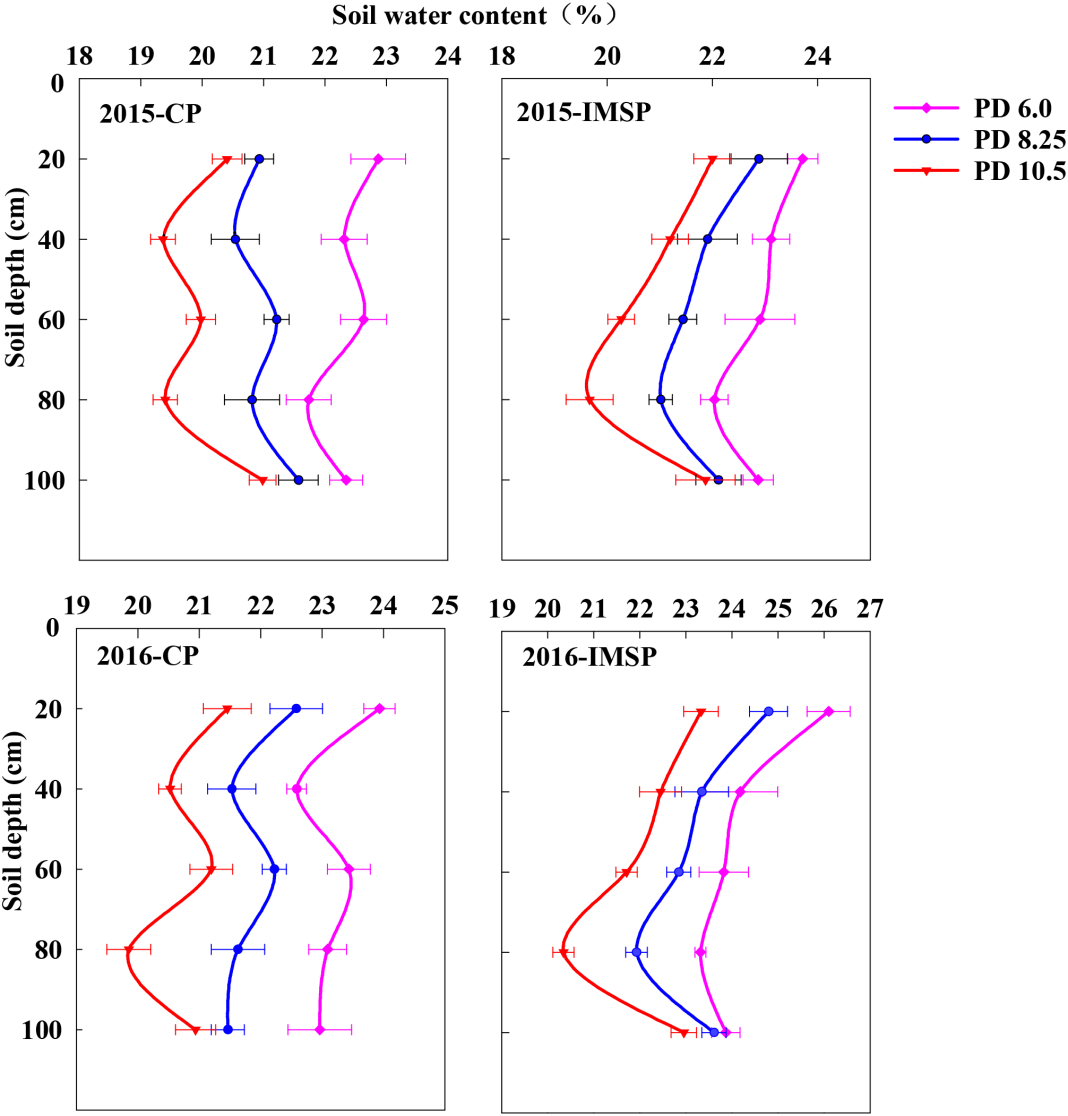
Changes of soil water content at silking stage under different soil management practices and plant densities in 2015 and 2016 study year.

The interaction between soil management mode and density had a significant effect on soil water content at silking stage (**Figure 5**). On the whole, the soil moisture content of IMSP mode is higher than that of CP mode, with an average difference of 26.6%. It mainly increased the water content of 0–40 and 80–100 cm soil layers. The comparison between different density populations showed that the high-density population aggravated the consumption of soil moisture. The IMSP mode not only promoted the absorption of soil moisture in 40–80 cm soil layer, but also significantly increased the gap between high-density PD10.5 and medium-low density, which was 25.3% larger than that of CP mode. It shows that the strong soil water supply capacity of IMSP mode is an important basis to support the strong soil water consumption of high-density population.

### 3.5 Effects of soil management, interaction of nitrogen application rate and density on WUE

**Figure 6:**
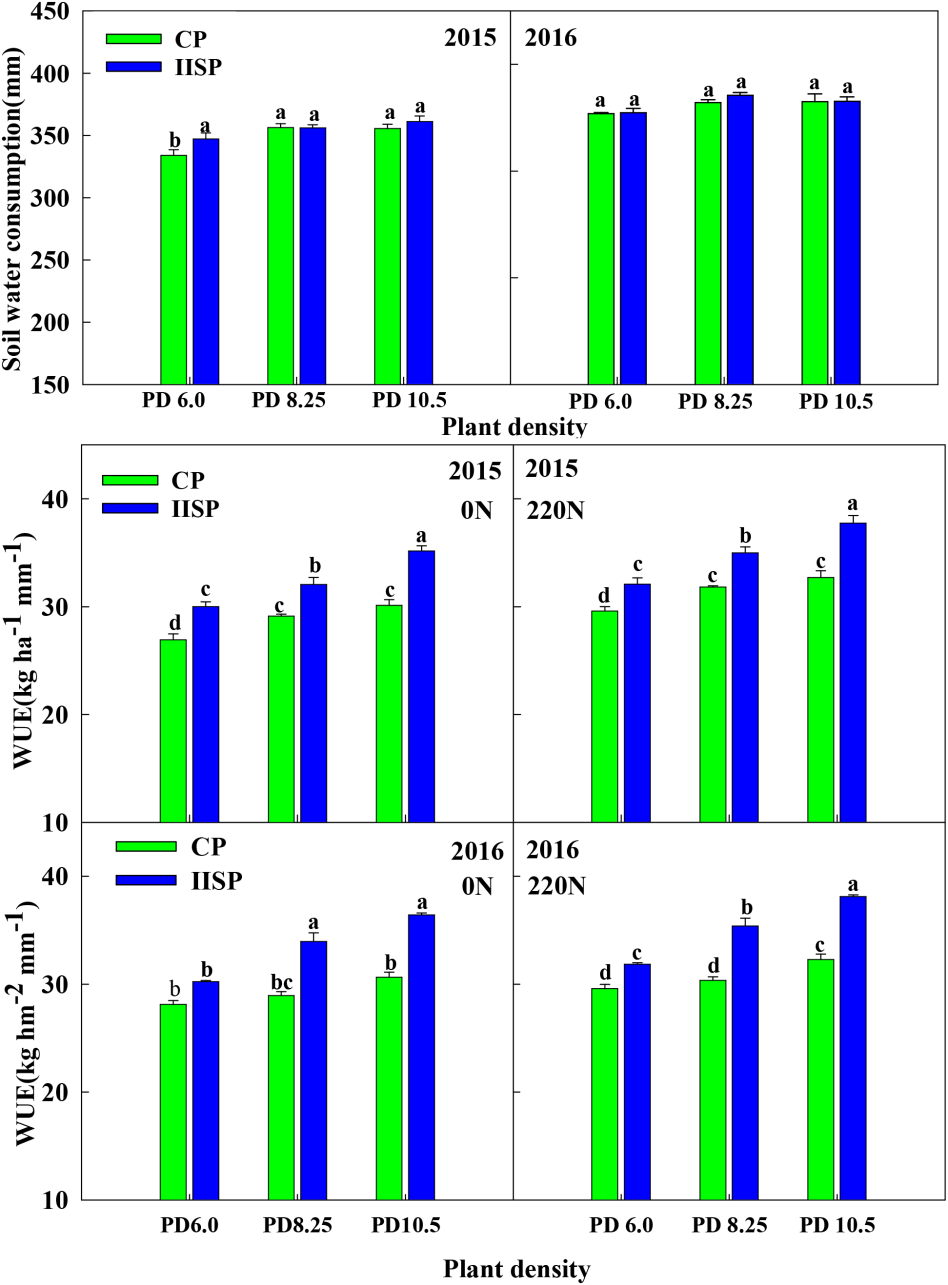
Comparison of water use efficiency soil and water consumption under different soil management practices、plant densities and nitrogen rates in 2015 and 2016 study year. Different lowercase letters indicate significant differences among treatments at P < 0.05.

It can be seen from **Figure 6** that the soil water consumption in 2016 was significantly higher than that in 2015, mainly due to the large rainfall during the growth period in 2016. The soil water consumption under IMSP mode was slightly higher than that under CP mode, but the difference was not significant. There was no significant difference in water consumption among different density populations.

**Figure 7:**
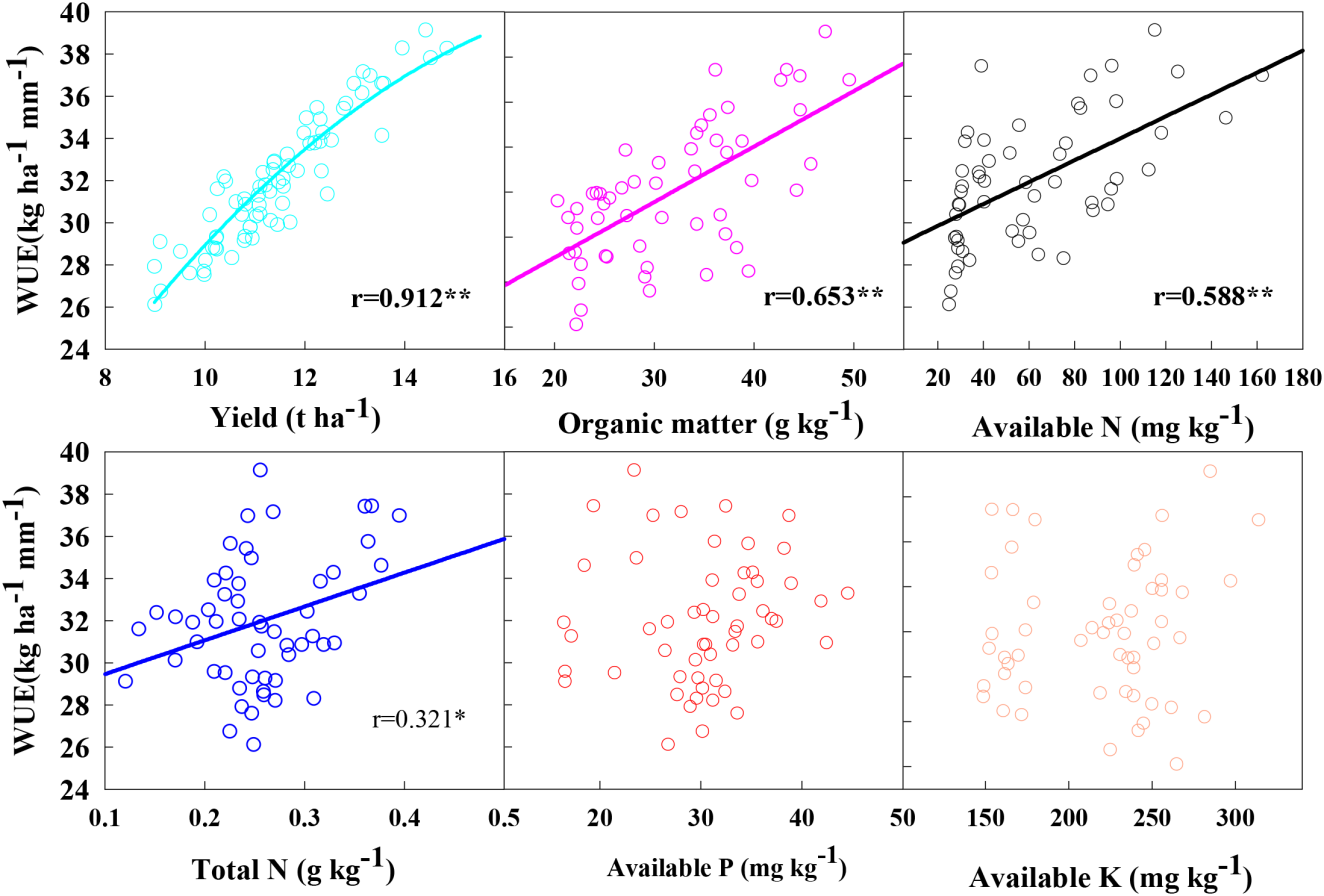
Correlations between water use efficiency and soil nutrient content. * and ** indicate significant differences at P < 0.05 and P < 0.01, respectively.

WUE is directly related to yield and water consumption during the whole growth period. As shown in **Figure 6**, IMSP significantly increased WUE by 12.9% on average compared with CP. Under IMSP mode, compared with low-density PD6.0, medium-density PD8.25 and high-density PD10.5 significantly increased WUE, while nitrogen application significantly increased the difference between high-density PD10.5 and medium-low density, with the highest difference of 19.7%. The correlation analysis results between WUE and other soil nutrient factors are shown in **Figure 7**. WUE was significantly positively correlated with soil organic matter, alkaline hydrolysis nitrogen and total nitrogen content (r=0.331*~0.687**), but the correlation between soil available phosphorus, available potassium and WUE was not significant. There was a significant positive correlation between yield and WUE (r=0.912**). That is to say, IMSP mode can improve WUE without increasing water consumption, which is closely related to the positive effect of soil organic matter, alkaline hydrolysis nitrogen and total nitrogen content on yield increase.

### 3.6 The relationship between soil nitrogen net mineralization, nitrogen apparent loss and yield under the interaction of soil management mode and density

**Figure 8:**
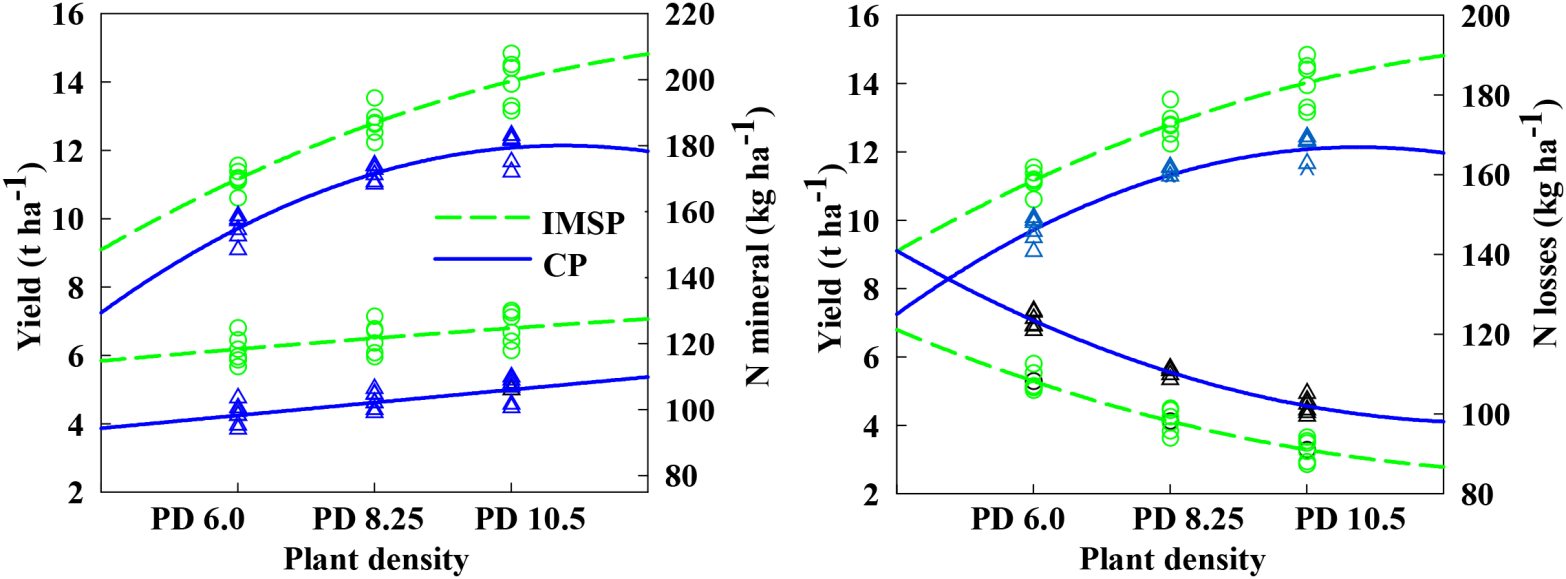
Relationship between nitrogen mineralization and nitrogen losses and yield under the interaction of soil management practice and plant density.

From **Figure 8**, it can be seen that the yield of the two soil management modes increases with the increase of density, but the difference is that the high-density population yield under the IMSP mode has not yet reached the peak. It shows that there is still room for further improvement in density and yield, and the yield of high-density population has tended to peak or even decreased significantly under CP mode. From the perspective of soil nitrogen mineralization characteristics, compared with CP mode, IMSP mode significantly increased the net mineralization of soil nitrogen, with an average difference of 16.0%. With the increase of density, the corresponding net mineralization of nitrogen increased slightly, indicating that the demand for nitrogen in the aboveground part stimulated soil nitrogen mineralization to a certain extent. In addition, the IMSP mode also significantly reduced the apparent loss of nitrogen, which was significantly reduced by 11.4% compared with the CP mode. Compared with the medium-low density, the apparent loss of nitrogen in the high-density PD10.5 population also showed a significant downward trend, with an average difference of 13.3% and a maximum difference of 17.4%. It can be seen that under the IMSP mode, the soil nitrogen mineralization ability and conservation ability were enhanced, and the apparent loss of soil nitrogen was significantly reduced. Therefore, the larger crop population received sufficient nitrogen supply, thus supporting the yield increase of the high-density population.

### 3.7 The relationship between population nitrogen uptake and soil nitrate nitrogen accumulation under the interaction of different soil management modes and nitrogen application rate and density

**Figure 9:**
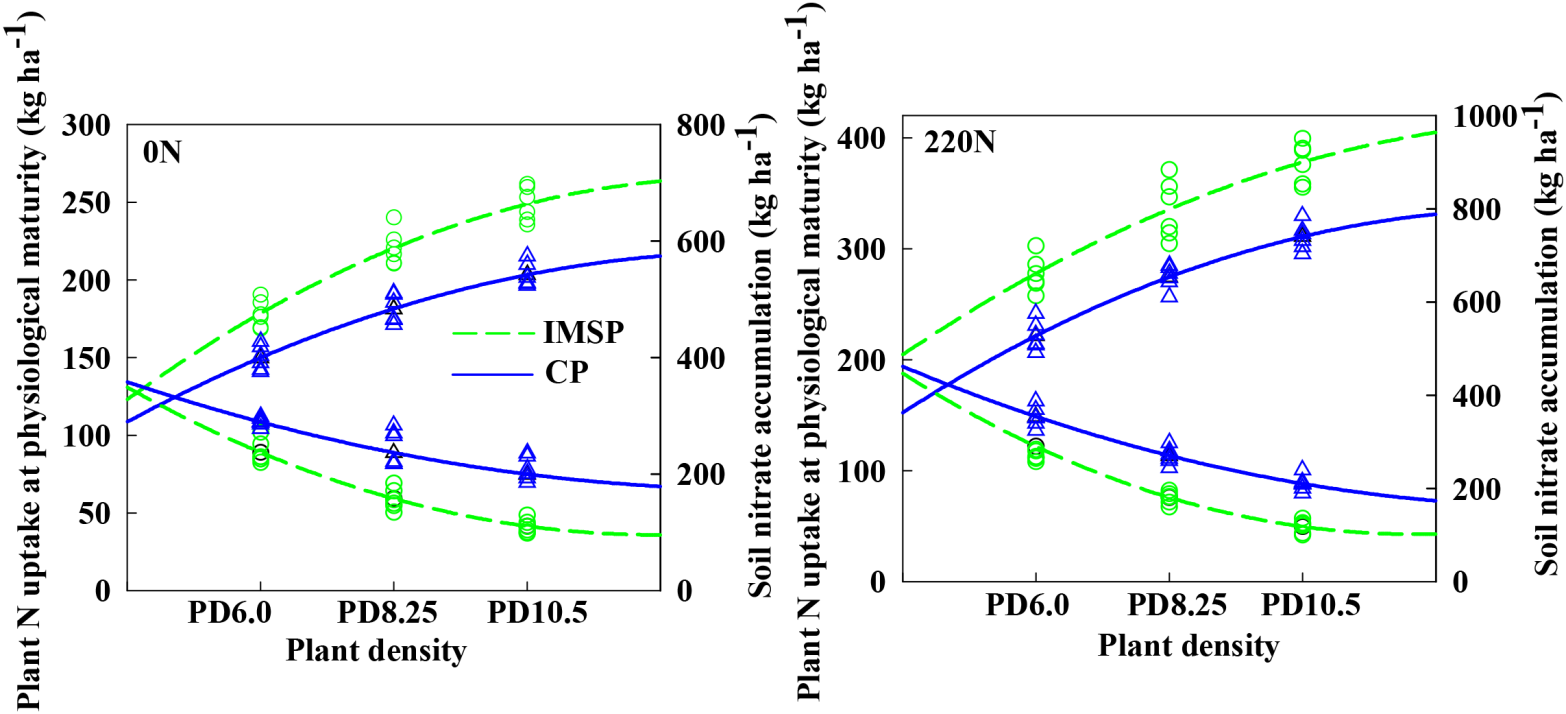
Correlations between plant N uptake at physiological maturity and NO_3_^--^ N accumulation under the interaction of soil management practice, nitrogen rate and plant density.

The interaction between soil management mode and nitrogen density had a significant effect on the relationship between plant nitrogen uptake and soil nitrate nitrogen accumulation (**Figure 9**). Compared with the CP mode, the IMSP mode significantly increased the population nitrogen accumulation, but the soil nitrate nitrogen accumulation at the mature stage was significantly reduced. Although nitrogen application showed similar rules, it significantly increased the gap between CP and IMSP modes. The total nitrogen accumulation increased significantly with the increase of density, while the soil nitrate nitrogen accumulation decreased significantly. The average difference between high-density 10.5 and low-density 6.0 in IMSP mode was 15.7% higher than that in CP mode. It can be seen that the IMSP model significantly promoted the absorption of soil mineralized nitrogen by the population, thus providing support for the absorption and utilization of nitrogen in the aboveground part.

## 4 Discussion

### 4.1 Key soil factors affecting inherent soil productivity, yield and NUE

Soil physical and chemical properties are often key determinants of soil productivity, although the dominant factors controlling inherent soil productivity may differ among farmland ecosystems (Amer et al., 2023). Analysis based on the boundary line method across 876 experimental sites in the central and southern wheat-growing region of Hebei Province indicated that soil pH, soil organic matter, and alkali-hydrolyzed nitrogen were the core factors influencing basic soil fertility (Huang et al., 2018). Moreover, total nitrogen and the C/N ratio were positively associated with stable inherent soil productivity (Huang et al., 2020). In this study, a random forest model was employed to quantitatively assess the importance of factors influencing changes in ISP, maize yield, and NUE. The results showed that Soil organic matter and alkali-hydrolyzable nitrogen were the main nutrient-related determinants of ISP, while soil water content, WUE, and N losses also contributed strongly, indicating that inherent soil productivity is jointly regulated by nutrient supply, water status, and N turnover. For maize yield, soil organic matter, alkali-hydrolyzable nitrogen, and available potassium were the major nutrient-related drivers, whereas N losses, WUE, and N mineralization showed the greatest contributions among the variables related to soil N transformation, physical structure, and water processes. A similar pattern was observed for NUE, with soil organic matter and alkali-hydrolyzable nitrogen remaining the key nutrient indicators, and N mineralization, N losses, and soil water content making major contributions. Overall, these results suggest that coordinated improvements in soil fertility, water availability, and N cycling are essential for enhancing productivity and NUE. This is basically consistent with the research results of Huang et al. (Huang et al., 2018).

### 4.2 Regulating soil moisture through soil inherent productivity to achieve the synergistic improvement of maize yield and NUE

The difference of soil water supply is embodied in the difference of soil bulk density and effective water content. As the core index of soil structure productivity, soil bulk density also has a good interaction with soil organic matter (Guo et al., 2016). Regulating soil organic matter can enhance soil aggregation, total porosity, hydraulic conductivity, water-holding capacity, water stability, and resistance to wind erosion, while reducing bulk density and compaction (Celik et al., 2004; Leroy et al., 2008; Martínez and Zinck, 2004). Compared with CP mode, IMSP mode significantly reduced soil bulk density, with an average difference of 1.4%. Moreover, under the IMSP mode, increasing planting density did not significantly increase soil bulk density, suggesting that the positive interaction between high soil fertility and planting density could alleviate soil compaction, improve soil aeration and water-holding capacity (Martínez and Zinck, 2004; Celik et al., 2010).

The IMSP mode improved the water retention capacity of each soil layer due to the interaction effect of straw returning and organic fertilizer, promoted the absorption of water in 40–80 cm soil layer by high-density population, stabilized the water in 0–40 cm soil layer, and effectively supplemented the water in 80–100 cm soil layer. It can be seen that the IMSP mode has a strong soil water supply capacity, which provides a guarantee for the large soil water consumption of high-density population. So, optimizing soil management measures can significantly improve soil biochemical properties, while improving yield and WUE (Xu et al., 2020). This was also confirmed in the present study, IMSP mode significantly improved WUE compared with CP mode, and densification and nitrogen application increased the difference between IMSP mode and CP mode. At the same time, the population water consumption of IMSP mode did not increase, indicating that the increase of yield under the soil fertility improvement mode greatly promoted the improvement of WUE, which was also closely related to the effective enhancement of soil water storage and conductivity and the reduction of water loss (Liu et al., 2025). The correlation analysis between WUE and soil nutrient factors showed that WUE was significantly positively correlated with soil organic matter, alkaline hydrolysis nitrogen and total nitrogen content. The mutual promotion of nutrient supply and water conditions made the soil fertility improvement model have a good soil water and nitrogen environment, which provided a strong guarantee for high yield and high efficiency of crops (Lu et al., 2011; Karami et al., 2012; Liu et al., 2013).

### 4.3 Regulating soil nitrogen through soil inherent productivity to achieve the synergistic improvement of maize yield and NUE

Coordinating and optimizing the balance between soil supply and crop demand is the key to increasing crop yield and efficiency (Chen et al., 2006; Li et al., 2014). The ability of crops to absorb inorganic nitrogen from soil is an important physiological process affecting yield formation (Sun et al., 2025). The difference of soil nitrogen supply capacity is also the core factor limiting the synergistic change of maize yield and NUE, and the difference of soil nitrogen supply can directly affect the formation of yield (Zhang et al., 2022). Meanwhile, microorganisms in high-fertility soils exhibit stronger nitrogen fixation capacity (Perelo et al., 2006; Said-Pullicino et al., 2014), which may help conserve potentially leachable nitrogen during periods of low plant demand and subsequently act as an available nitrogen source at later growth stages (Choi et al., 2004; Herai et al., 2006; Sugihara et al., 2012), thereby exerting a stimulatory effect on fertilizer nitrogen use efficiency. The results of this study showed that the IMSP model significantly increased the net mineralization of soil nitrogen, which was 16.0% higher than that of the CP model, and the N losses were 11.4% lower than that of the CP model. The difference of N losses between high and low densities was large, and the highest difference was 17.4%. This is basically consistent with the research results of Cao B et al. (Cao et al., 2022). He believes that high soil fertility farmland can have strong soil nitrogen supply capacity, which may be closely related to soil organic matter content. At the same time, based on the inherent soil productivity of ISP 8.0 t ha^-1^, this study compared the content of soil nutrient core limiting factors of organic matter and alkaline hydrolysis nitrogen. It was found that there was a significant difference in alkaline hydrolysis nitrogen above and below this critical point, and the content of soil organic matter also showed a significant difference at the lower limit of the critical point, and the content of organic matter was significantly positively correlated with alkaline hydrolysis nitrogen and soil nitrogen net mineralization. That is to say, when the soil organic matter content is increased, the soil available nitrogen, nitrogen mineralization ability and water conservation ability can be indirectly affected, thereby improving the soil fertilizer supply capacity.

The mismatch between nutrient demand and soil nutrient supply in high-density planting population is an important internal factor limiting the coordinated development of high yield and high efficiency (Zhao et al., 2019). The results of this study showed that through the IMSP soil management mode, the yield of high-density population still had room for further improvement, but under the CP mode, the yield would have a significant downward trend. This is mainly attributed to the fact that the IMSP model expands the soil nitrogen storage capacity and can provide sufficient nitrogen for high-density populations, thereby supporting high-density populations to further increase production, which is also an important internal factor for the increase of NUE after improving inherent soil productivity (Du et al., 2024). The absorption and accumulation of nitrogen by crops is premised on the consumption of nitrogen in the soil, especially nitrate nitrogen. This study also found that IMSP significantly promoted the absorption of mineralized nitrogen by the population compared with CP mode, and reduced the residual nitrate nitrogen in the mature period. That is to say, the soil fertility improvement model can effectively reduce the risk of nitrogen leaching, and improving inherent soil productivity is an important basis for achieving high yield, high efficiency and environmental friendliness of maize. Studies have shown that compared with low-medium fertility farmland, high-fertility farmland has enough nutrient resources to match crop growth needs in real time, High-fertility farmland formation could not only retain organic N but also affect the availability of soil N by regulating soil nitrification potential (Wang et al., 2019). In this study, different yield and NUE groups were created by deep plowing combined with soil fertility improvement technology with organic fertilizer and nitrogen-density interaction. The results showed that the IMSP model and CP model affected maize yield and NUE synergistic changes mainly in the difference of soil water supply and fertilizer supply capacity.

## 5 Conclusions

Soil organic matter and alkali-hydrolysable nitrogen were the major nutrient-related drivers of ISP, maize yield, and NUE, whereas N mineralization, N losses, soil water content, and WUE dominated process-related regulation. Among these, soil organic matter was the core factor governing ISP. The core of synergistic regulation of maize yield and NUE by soil fertility improvement is mainly manifested in two aspects: on the one hand, it meets the demand of maize population for water and nutrients by expanding the storage capacity of soil moisture and nutrients; on the other hand, by increasing soil nitrogen mineralization (16.0%) and reducing nitrogen loss (11.4%), the soil has a better soil water and nitrogen environment, thus affecting yield and NUE. The interaction between IMSP mode and nitrogen density had a more significant regulatory effect on increasing yield and efficiency. Therefore, the synergistic improvement of yield and NUE through soil fertility improvement needs to combine nitrogen-density interaction to exert its comprehensive effect.

## Data Availability

The original contributions presented in the study are included in the article/**Supplementary Material**. Further inquiries can be directed to the corresponding author/s.
