# Supplementary material for: Enhancing inherent soil productivity increases maize yield and nitrogen use efficiency by improving soil water and nutrient status
Source: Front Plant Sci. 2026 Apr 14;17:1816672. doi: 10.3389/fpls.2026.1816672 (PMC13121264; doi:10.3389/fpls.2026.1816672)
Supplement: Supplementary file 1 [file DataSheet1.docx]

**Enhancing inherent soil productivity increases maize yield and nitrogen use efficiency by improving soil water and nutrient status**

**Zhipeng Cheng****^1, 2^****^, †^, Meiren Rong^1, 2†^, Yajian Li^1, 3^, Fugui Wang^1, 2^, Zhen Wang^1, 2^, Yongqiang Wang^1, 2^, Ranran Guo^1, 2^, Lanfang Bai^1, 2,^ *, Zhigang Wang^1, 2^***

^1^ College of Agronomy, Inner Mongolia Agricultural University, Hohhot 010019, China;

[chengzhipeng@emails.imau.edu.cn(Z.C.)](mailto:chengzhipeng@emails.imau.edu.cn(Z.C.)); [lfbai@imau.edu.cn(L.B.)](mailto:lfbai@imau.edu.cn(L.B.)); l[iyajian.fly@163.com(Y.L)](mailto:iyajian.fly@163.com(Y.L)); nmgwfg@imau.edu.cn (F.W.); [cau1022@imau.edu.cn](mailto:cau1022@imau.edu.cn) (Z.W.); [wangyongqiang@nwafu.edu.cn](mailto:wangyongqiang@nwafu.edu.cn) (Y.W.); [guoranran@emails.imau.edu.cn(R.R.)](mailto:guoranran@emails.imau.edu.cn(R.R.)); [rongmeiren@emails.imau.edu.cn](mailto:rongmeiren@emails.imau.edu.cn) (M.R.);

^2^ Inner Mongolia Autonomous Region Industrial Technology Engineering Center for Intelligent Water and Fertilizer Management Technology and Equipment for Crops, Hohhot 010018, China

^3^ Institute of Biotechnology, Tongliao Academy of Agricultural and Animal Husbandry Sciences, Tongliao, China

***** Correspondence: zgwang@imau.edu.cn (Z.W.)

^†^ Contributed equally to this work and are co-first authors.

Table S1. Locations, soil fertility, and weather data from April to October of the multisite-year network experiment conducted in Inner Mongolia, China.

| Ecological region | Trial location | |  |  |  |  | Soil fertility | | | |
| --- | --- | --- | --- | --- | --- | --- | --- | --- | --- | --- |
|  | Latitude | Longitude | Soil  texture | Year | Average temperature  (℃) | Precipitation  （mm） | Soil organic  Matter (g kg^−1^) | Available N  (mg kg^−1^) | Olsen P  (mg kg^−1^) | Available K  (mg kg^−1^) |
| Hilly region of southern  greater Khingan | 46°45′N | 122°47′E | Sandy clay  loam | 2015 | 17.6 | 436.9 | 16.2 | 80.3 | 54.7 | 108.4 |
|  |  |  |  | 2016 | 20.2 | 378.8 | 17.9 | 101.4 | 31.2 | 164.9 |
| West Liao River Plain | 43°44′N | 122°32′E | Silty loam | 2015 | 20.5 | 433.2 | 20.3 | 65.6 | 28.2 | 267.9 |
|  |  |  |  | 2016 | 21.3 | 331.6 | 21.1 | 77.3 | 32.8 | 227.4 |
| Hilly region of  northern Yan  mountains | 42°18′N | 118°10′E | Sandy loam | 2015 | 19.1 | 273.1 | 21.7 | 59.2 | 29.4 | 113.8 |
|  |  |  |  | 2016 | 20.2 | 501.7 | 22.9 | 53.9 | 34.8 | 151.7 |
| Tumed Plain | 40°32′N | 110°28′E | Silty loam | 2015 | 17.8 | 275.4 | 26.7 | 77.1 | 24.1 | 106.3 |
|  |  |  |  | 2016 | 19.6 | 308.2 | 29.7 | 100.1 | 18.3 | 151.0 |
| Hetao Plain | 41°11′N | 108°49′E | Clay loam | 2015 | 17.0 | 230.5 | 18.2 | 79.5 | 24.5 | 214.5 |
|  |  |  |  | 2016 | 18.2 | 138.5 | 16.4 | 108.0 | 34.6 | 195.6 |

Table S2. Inherent soil productivity and maize grain yield of treatments averaged at the five ecological regions in 2015 and 2016 (kg ha^−1^)

| Ecological region |  | Hilly region of southern greater  Khingan | West Liao River Plain | Hilly region of northern Yan mountains | Tumed plain | Hetao plain |
| --- | --- | --- | --- | --- | --- | --- |
| ISP |  | 7.6 | 11.5 | 10.3 | 8.0 | 10.2 |
| Soil management (S) | CP | 9.3 | 14.8 | 10.4 | 11.2 | 11.4 |
|  | IMSP | 10.3 | 15.1 | 12.1 | 12.3 | 13.1 |
| Plant density (PD) | 6.0 | 9.6 | 13.2 | 10.7 | 10.2 | 11.3 |
|  | 8.25 | 10.2 | 14.8 | 11.3 | 13.0 | 12.1 |
|  | 10.5 | 9.7 | 16.8 | 11.7 | 12.0 | 13.3 |
| N Rate (N) | 0 | 8.3 | 12.5 | 11.1 | 8.5 | 11.1 |
|  | 220 | 9.8 | 14.9 | 11.3 | 11.8 | 12.3 |
| Source of variance |  | | | | | |
| S |  | ** | ** | ** | * | ** |
| PD |  | NS | ** | ** | * | ** |
| N |  | ** | ** | ** | ** | ** |
| S×PD |  | NS | NS | NS | NS | ** |
| S×N |  | NS | NS | * | NS | NS |
| PD×N |  | NS | ** | NS | * | * |
| S×PD×N |  | NS | NS | ** | NS | ** |

Note: * indicates significance at the 0.05 level; ** indicates significance at the 0.01 level; NS indicates non-significance.
